# Does hybridized dentin affect bond strength of self-adhesive resin cement?

**DOI:** 10.4317/jced.52980

**Published:** 2016-10-01

**Authors:** Saulo Pamato, Accácio-Lins do Valle, Gustavo-Henrique-Barbosa de Andrade, Hugo-Alberto Vidotti, Marcus-Vinícius-Reis Só, Jefferson-Ricardo Pereira

**Affiliations:** 1 DDS, MSc, Program of Health Science, University of Southern Santa Catarina, Tubarão, Santa Catarina, Brazil; 2 DDS, PhD, Department of Prosthodontics, University of São Paulo, Bauru, São Paulo, Brazil; 3 DDS, MSc, Department of Prosthodontics, University of São Paulo, Bauru, São Paulo, Brazil; 4 DDS, PhD, Department of Endodontic, Federal University of Rio Grande do Sul, Porto Alegre, Rio Grande de Sul, Brazil; 5 DDS, PhD, Program of Health Science, University of Southern Santa Catarina, Tubarão, Santa Catarina, Brazil

## Abstract

**Background:**

Evaluate the influence of different hybridization bonding techniques of a self-adhesive resin cement.

**Material and Methods:**

30 human health molars were divided into six groups (n=10). The specimens received three longitudinal sections, allowing insertion of central cuts in PVC matrices. Each group received a different dentin pretreatment according to the manufacturer’s recommendations, except the control group (G1), as follows. G2 - a 3-step total-etch adhesive system (Optibond™ FL, Kerr); G3 - a 3-step total-etch adhesive system (Adper™ Scotchbond™ Multi-Purpose, 3M ESPE); G4 - a 2-step total-etch adhesive system (Adper™ Single Bond 2, 3M ESPE); G5 - a single-step self-etching system (Bond Force, Tokuyama); and G6 - universal bonding system (Single Bond Universal, 3M ESPE). Then, cylinders made of self-adhesive resin cement with polypropylene matrix was cemented in all groups (RelyX U200, 3M ESPE). Bond strength was assessed by submitting the specimens to micro-shear test and was characterized according to the fracture pattern observed through optical microscopy.

**Results:**

The results were submitted to the Kruskal-Wallis test, which indicated a statistically significant difference between the groups (*p*=0.04), and Tukey’s multiple comparisons, which indicated a statistically significant difference between G1 and G3 (*p*<0.05). The microscopic analysis revealed a high prevalence of adhesive failures, followed by mixed fractures, and cohesive failures in the dentin.

**Conclusions:**

The use of a previous dentin hybridization protocol is able to increase adhesive bonding resistance of self-adhesive resin cement, especially when used Adper™ Scotchbond™ Multi-Purpose system.

** Key words:**Bonding, self-adhesive resin cement, adhesive systems, microshear.

## Introduction

The increasing demand for highly physical and aesthetic tooth restorations has spurred research and development of materials that reproduce the shape, color, texture and function of missing natural teeth. Depending on the evolution of large ceramic coating, new restorative systems could be developed, such as those reinforced by alumina, leucite, lithium disilicate, and zirconia (1). In contrast, the absence of a cementitious material with ideal characteristics sometimes compromised the success of the definitive rehabilitative treatment, and become a constant concern among researchers and clinicians throughout the ages (2).

The studies on enamel etching by Buonocore (3) (1995) and the composite-resin systems by Bowen (4) (1963) resulted in the development of resin-based cements. Considering the treatment of the structures involved in the process, resin-based cements could ensure bonding to both the tooth structure and the dental prosthesis, resulting in the formation of a single body, which allowed for a better distribution of loads during chewing and lower fracture risk, thus preventing premature microleakage (5).

The bonding principle of resin cements to tooth tissues consists of the removal of calcium phosphate, which facilitates the creation of micro porosities by conditioning the surface, and subsequently, infiltration and polymerization of the resin within these spaces, ensuring a micromechanical lock based on the diffusion principle (6). However, sensitivity of the adhesive technique and its interaction with the resin cements require considerable knowledge of the operator to show such properties (7).

In recent years, other cementing materials have been made available in the dental market in order to produce adhesion through a simple application protocol, as an alternative to the systems currently used for cementation (8). These cements have a self-adhesive characteristic; they do not require total-etch and washing, and are able to promote surface demineralization of the substrate and penetration into the tooth structure through its initial acidity (pH=2).

Little information exists about the composition and the bonding mechanism of adhesive cements. Thus, the objective of this study was to evaluate the influence of different hybridization techniques on the immediate resin-bond strength of self-adhesive resin cements.

## Material and Methods

This study was approved by the Research Ethics Committee of the University of Southern Santa Catarina (UNISUL). Thirty freshly extracted caries-free human molars with similar dimensions and anatomic structure obtained from the UNISUL tooth bank were used in this study. Those with no cleavage or visible cracks were selected and properly stored in distilled water throughout the experiment.

The teeth were cleaned from remnant soft tissue and stored in 0.5% chloramine T at room temperature during the first 7 days after extraction and thereafter stored in distilled water at 5˚ C for a maximum of 6 months.

Three standardized longitudinal sections were made on each specimen using a low-speed diamond disk under water-cooling in the cutting machine (ISOMET® 1000, Buehler, Lake Buff, IL, USA), in order to split it into 4 parts as follows: buccal, midbuccal, midlingual, and lingual. The two central sections of each specimen were embedded in acrylic resin (JET, Classico, São Paulo, SP, Brazil), using a matrix of rigid PVC (AMANCO, São Paulo, SP, Brazil), and were smoothed and polished with (Arotec® APL-4, São Paulo, SP, Brazil) polisher and silicon carbide paper in decreasing order of grain size (#200, #400, #600, and #1200), and aluminum oxide paste (Diamond R, FGM, Joinville, SC, Brazil).

**Figure 1:**
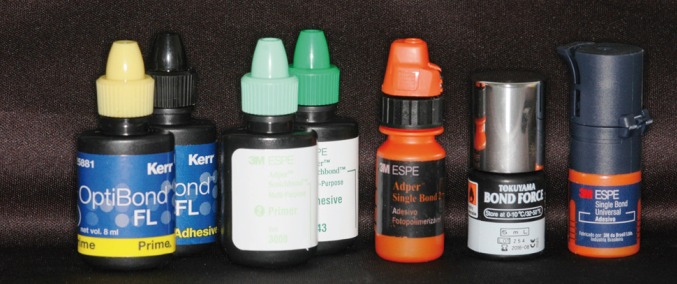
Adhesive systems used in the experiment.

The specimens were randomly divided into 6 groups (n=10), according to the different type of conditioning they received: G1 - no surface conditioning (control), G2 – three-step total-etch adhesive (OptiBond™ FL, Kerr, Orange, CA, USA), G3 - three-step total-etch adhesive (Adper™ Scotchbond™ Multi-Purpose, 3M ESPE, St Paul, MN, USA), G4 - two-step total-etch adhesive (Adper™ Single Bond 2, 3M ESPE, St Paul, MN, USA), G5 – one-step self-etch adhesive system (Bond Force, Tokuyama, Osaka, Japan), G6 - Universal system (Single Bond Universal, 3M ESPE, St Paul, MN, USA) (Fig. 1).

**Figure 2:**
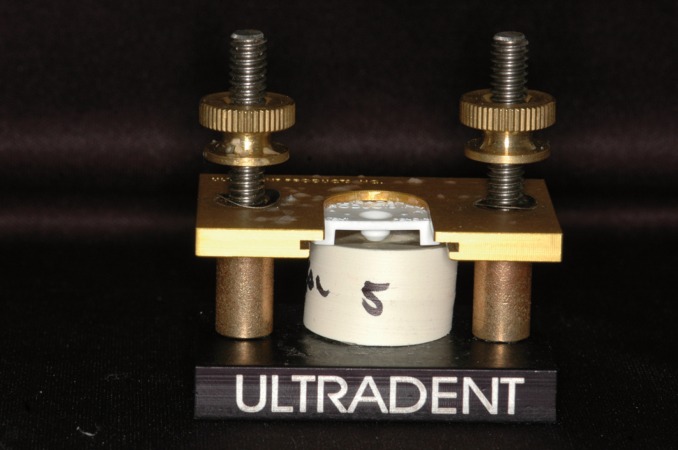
Polypropylene matrix attached to the metal device used for the fabrication of self-adhesive resin cement cylinders.

After dentin hybridization was completed, a cylinder of self-adhesive resin cement (RelyX™ U200, 3M ESPE, St Paul, MN, USA) was placed near the cement-enamel junction (CEJ), perpendicular to the exposed dentin, using a polypropylene matrix with a 2.3 mm center hole (Bonding Mold Inserts, Ultradent, South Jordan, UT, USA) attached to a metal device (Bonding Clamp, Ultradent, South Jordan, UT, USA), as shown in figure 2. A lentulo drill (Malleiffer, Ballaigues, Switzerland) was used in a contra-angle handpiece. The center hole was filled with resin cement and light cured for 40 seconds using an LED curing light (Valo®, Ultradent, South Jordan, UT, USA).

**Figure 3:**
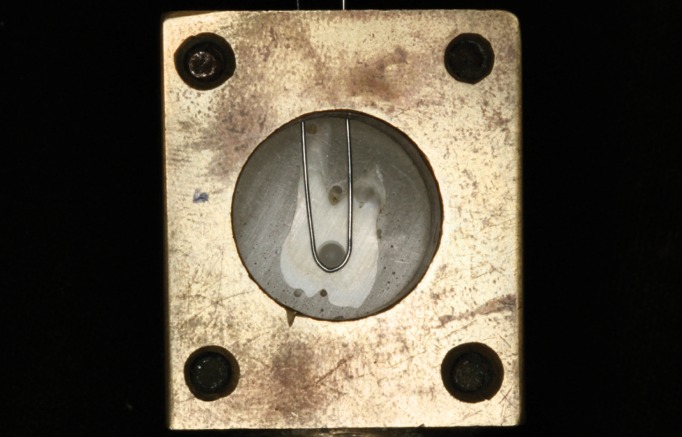
A specimen attached to the device and laced with wire.

The cell samples were adapted into the metallic device with accurate alignment of the load string of a universal testing machine (EMIC DL2000, São José dos Pinhais, PR, Brazil), and the cylinder was fasted using a wire 0.2 mm in diameter parallel to the exposed face of the tooth (Fig. 3). Then, a shear stress of 0.5 mm/min was applied until the fracture occurred. At the end, the values recorded in Newton force (N) by the Tesc software (Tesc 3.04, EMIC, São José dos Pinhais, PR, Brazil) were converted to MegaPascal (MPa) as follows: MPa = Newton/surface area (mm2).

All specimens subjected to microshear testing were prepared for the analysis of fracture pattern under a stereomicroscope (Stemi DV4, Zeiss Universal Microscope, Jena, Germany) and optical microscope (N107, Coleman, Santo André, SP, Brazil) at 40X magnification. The fracture patterns were then classified into the following: 1) adhesive fracture: fracture at the bond interface; 2) cohesive fracture in the dentin: complete break within the dentin; 3) cohesive failure of the cement: complete disruption of the cement cylinder; 4) Mixed fracture: breaking involving two or more different tooth substrates.

Kruskal-Wallis and Tukey tests were used to examine statistically significant differences between groups. The significance level was set at 5%.

## Results

The Shapiro-Wilk test of normality was used for data analysis, which indicated the need for a non-parametric test (*p*<0.05) in determining statistical differences between groups. The Kruskal-Wallis test (*p*=0.05) was used when there was a single factor (dentin).

The Kruskal-Wallis test results identified statistically significant differences between the tested groups (*p*=0.04). In order to determine such differences, the Tukey’s multiple comparison test was used. The results are shown in  Table 1.

**Table 1:**
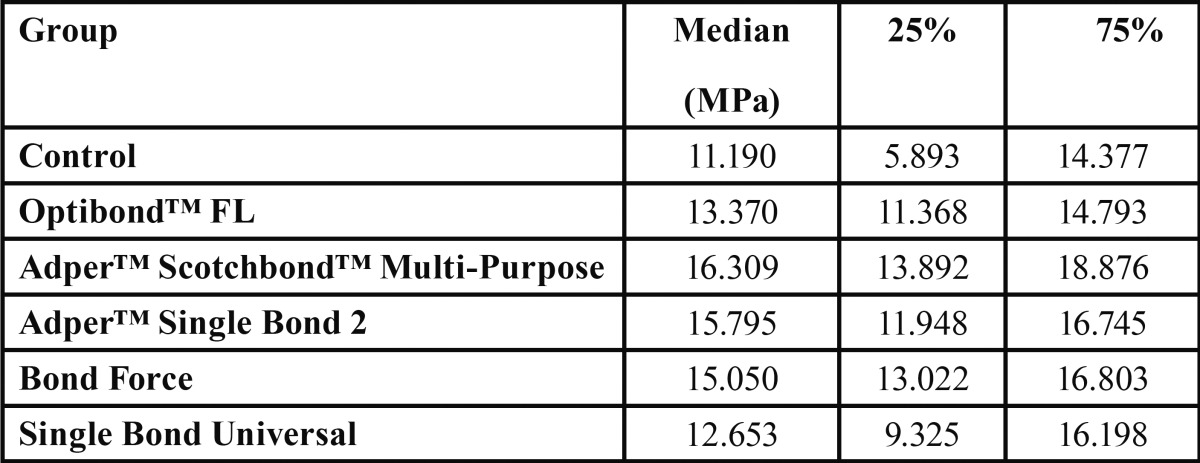
Groups, median, and upper and lower quartile values.

With regard to the type of fracture analysis, which was performed using optical microscopy, the data are displayed by group and by the presence or absence of intervention ( Table 2).

**Table 2:**
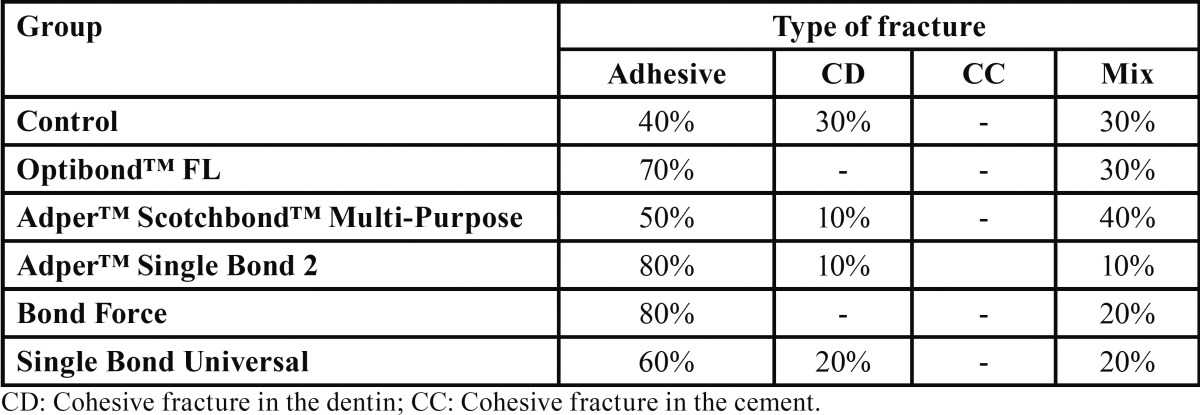
Type of fractures per group.

## Discussion

Based on the results of this study, dentin hybridization with different dentin bonding systems showed distinct performances according to the luting agents and the techniques used. Whereas some groups did not differ statistically from the control group, the specimens hybridized with Adper™ Scotchbond™ Multi-Purpose (3M ESPE) had significantly higher microshear bond strength. Thus, the null hypothesis, which stated that dentin hybridization would not improve bond strength of adhesive resin cements was rejected.

Self-adhesive resin cements were introduced in the market with the purpose of eliminating the substrate pretreatment required in conventional resin cements. However, it is known that bonding interface quality is closely associated with the extent of resin monomer infiltration of the previously demineralized collagen mesh. Despite its low initial pH, high viscosity of adhesives prevents surface wetting and infiltration into tooth substrates (9). These principles explain the findings of this study, showing that surface conditioning by using a potent agent, such as phosphoric acid, followed by the application of a fluid-bonding agent, is able to promote an increase in bond strength of adhesive resin cements.

Conventional adhesive systems are widely considered in dentistry, and have shown good results in clinical and laboratory tests. The difference in the scores achieved by such systems in the present study reinforces the thesis that high-tensile bond strength is not the only criterion for a successful bonding process. This can rather be attributed to the superior quality of the adhesive interface, as well as an ideal relationship between adhesive thickness and bond strength (10). However, it is noteworthy that our findings do not confirm those published by Pashley *et al.* (11) (2011), since the Optibond™ FL system containing ethanol as solvent in the formulation of the primer showed the lowest bond strength when compared to Adper™ Scotchbond™ Multi-Purpose system. According to the authors of that study, the presence of ethanol in the solvent would be capable of promoting a chemical dehydration of the demineralized collagen matrix, resulting in a lateral shrinkage of the collagen fibers, an increase in the width of interfibrillar spaces, and therefore, a reduction in the hydrophilic content of the collagen matrix.

According to Pashley *et al.* (11) (2011), the longevity of the current restorative procedures is still below that guaranteed by the amalgam fillings, since the dentin bonding strength protocol does not have the same tensile bond strength on enamel. When compared to the simplified system, the results in this study were not different from those obtained more than 10 years ago by De Munck *et al.* (12) (2003). This fact could explain the significant reduction in bond strength of this system because of the high hydrophilic content of the formula components that prevent adequate penetration of adhesive monomers into the wet collagen matrix, greatly compromising the properties of the hybrid layer. The literature is concise and unanimous in stating that bond strength of resin-based cements is less effective than that of the adhesive systems used in direct composite resin restorations (5,13,14). Since mid-2003, the self-etch adhesive systems have been elected as excellent adjuncts to improve the performance shown by the adhesive resin cements, mainly by increasing bond strength and because of low technique sensitivity (15).

However, those data do not confirm the results obtained in this study, even in dentin tissue, which showed no statistically significant difference between the self-etch adhesives and the control group. This divergence can be explained either by the considerable evolution in the composition of the adhesive resin cements over the 10 years or by the increase in hydrophilic properties of self-etch systems, which is a relevant factor that influences polymerization, capable of allowing an uninterrupted substrate conditioning and its final adhesive capacity (16).

Some studies have highlighted the importance of the MDP hydrophilic acid monomer in the composition of self-etch adhesive systems, and recently, single bond universal adhesive system (17-19). According to those authors, such a high molecular weight component is able to promote an ionic bond to hydroxyapatite through the low solubility of the calcium salt on its surface, which organize themselves into highly hydrophobic nano-layers, thus protecting the hybrid layer of hydrolytic degradation (20). Accordingly, in face of the rehydration possibility of collagen fibers after being dried by the presence of the copolymer Vitrebond™, such a system would undoubtedly become the gold standard. However, it showed tensile bond strength values very close to the other hybridized groups, and there were no significant statistical differences that justify its use prior to cementing procedures.

With regard to the type of failure analysis, the study results showed high prevalence of adhesive ruptures (63.3%), followed by mixed failures (25%) and cohesive fracture in the dentin (11.7%), which supports the findings by Hori and De Carvalho (21) (2012) . It was supposed that the low tensile bond strength and adhesive bonding were sufficient to prevent fractures on the self-adhesive resin cement cylinder. However, these data do not support the findings by Oilo and Austrheim (22) (1993) who advocated that conventional studies using shear methodology presented, in most cases, cohesive failures in the dentin, not representing the bond strength to dentin of these luting agents.

The microtensile bond test was initially proposed by Sano *et al.* (23) (1994), and it is currently used in more than sixty percent of the published studies. When compared to macro tests, the microtensile bond tests have advantages, such as a better use of specimens, greater control in determining the working area, better stress distribution, among others. In contrast, those authors emphasized that when the cuts are not performed meticulously, the interface defects can easily compromise the specimens, resulting in a premature fracture point. For that reason, trainings of the operator and standardization of the specimens in setup tests are essential for obtaining reliable results. Introduced in 2002, microshear tests combine ease of handling and capability of testing multiple specimens per tooth (24), which allows for the application of a force on a body built perpendicular to the substrate in question. However, the small size of this body can either make it impossible to timely implement a bonding system, or allow its bending in the presence of non-uniformed forces.

The technical simplicity of the shear tests alone should not justify their use in the evaluation of bond strength. It is noteworthy that, numerically, microshear values reach one-third of microtensile scores. However, they do not show differences in the failure pattern (25).

As mentioned earlier, *in vitro* tests use simplified methods when compared to *in vivo* situations. In the oral environment, teeth are constantly subjected to different types of stress, which can cause failures that are identified through mechanical tests, such as pH changes, occlusal loading, and enzymatic challenges (18). The results of this study indicated the need for long-term research that can clarify the relationship between contemporary bonding systems and self-adhesive resin cements.

Based on the results and considering the limitations of this *in vitro* experiment, this study concluded that prior dentin hybridization does not negatively interfere with the bond strength of self-adhesive resin cement. Among the various systems and techniques used, the Adper™ Scotchbond™ Multi-Purpose adhesive showed the best bonding results, being the only adhesive system that significantly differed from the control group.
